# Draft Genome Sequences of a Phylogenetically Diverse Suite of *Pseudomonas syringae* Strains from Multiple Source Populations

**DOI:** 10.1128/genomeA.01195-13

**Published:** 2014-01-23

**Authors:** David A. Baltrus, Scott Yourstone, Abigail Lind, Caroline Guilbaud, David C. Sands, Corbin D. Jones, Cindy E. Morris, Jeffrey L. Dangl

**Affiliations:** a School of Plant Sciences, University of Arizona, Tucson, Arizona, USA; b Department of Biology, University of North Carolina at Chapel Hill, Chapel Hill, North Carolina, USA; c INRA, UR0407 Pathologie Végétale, Montfavet, France; d Plant Sciences and Plant Pathology Department, Montana State University, Bozeman, Montana, USA; e Carolina Center for Genome Sciences, University of North Carolina at Chapel Hill, Chapel Hill, North Carolina, USA; f Howard Hughes Medical Institute, University of North Carolina at Chapel Hill, Chapel Hill, North Carolina, USA

## Abstract

Here, we report the draft genome sequences for 7 phylogenetically diverse isolates of *Pseudomonas syringae*, obtained from numerous environmental sources and geographically proximate crop species. Overall, these sequences provide a wealth of information about the differences (or lack thereof) between isolates from disease outbreaks and those from other sources.

## GENOME ANNOUNCEMENT

*Pseudomonas syringae* is well known as a facultative phytopathogen of many plant species, including crops, such as tomato, bean, and wheat, as well as the laboratory model *Arabidopsis* (1). Therefore, a majority of *P. syringae*-related research has focused on identifying and understanding the virulence factors that enable this bacterium to cause disease *in planta*. More recently, increased emphasis has been placed on understanding how *P. syringae* strains survive in the environment outside plants, and greater environmental sampling has driven the collection of strains from noncrop sources and symptomless plants (2–4). Here, we describe the draft genome sequences for phylogenetically diverse isolates of *P. syringae* collected from various sources in the United States and France. The sampled locations include nonagricultural environmental sources as well as plants lacking disease symptoms. These sequences provide a wealth of data for genomic comparisons between geographically similar isolates and enable a greater understanding of the evolutionary forces that lead to the emergence of phyopathogens from environmental reservoirs.

The collection of these isolates has been previously described (3–5). Genomic DNA was prepared from populations initiated with single colonies and purified as per Baltrus et al. (6). The sequencing of each isolate except *P. syringae* USA011 was performed on an Illumina GAII from 36-bp paired-end libraries. A genomic library from USA011 was sequenced using a portion of one Illumina HiSeq lane using 36-bp paired-end libraries. The draft genomes were assembled using SPAdes 2.5.0 without trimming for quality (7). These assemblies are part of a larger project that includes draft genome assemblies for *P. syringae* strains (and their respective accession no.) UB246 (AVEQ00000000), CC1543 (AVEJ00000000), CC94 (AVEA00000000), UB303 (AVDZ00000000), CC1416 (AVEP00000000), CC1458 (AVEN00000000), CC1544 (AVEI00000000), CC1559 (AVEG00000000), CC440 (AVEC00000000), CC457 (AVEB00000000), CC1557 (AVEH00000000), USA007 (AVDY00000000), and CC1583 (AVEF00000000), as well as *P. viridiflava* strains CC1582 (AVDW00000000) and TA043 (AVDV00000000). These assemblies are not included within this announcement due to poor assembly quality.

### Nucleotide sequence accession numbers.

The nucleotide sequence accession numbers for Genbank are found in Table 1. These sequences and associated metadata can be publicly found at the Joint Genome Institute (JGI) portal (http://img.jgi.doe.gov/) with the JGI taxon IDs listed in Table 1.

**TABLE 1:** Characteristics of the seven *P. syringae* isolates

Isolate	NCBI accession no.^*a*^	JGI taxon ID	Genome size (bp)	No. of contigs	N_50_	Substrate of isolation	Country of isolation	Reference
*Pseudomonas syringae* CC1417	AVEO00000000	2506783030	5,648,464	210	50,166	Epilithon	USA	3
*Pseudomonas syringae* CC1466	AVEM00000000	2506783019	5,591,749	294	60,479	*Dodecatheon pulchellum*	USA	3
*Pseudomonas syringae* CC1513	AVEL00000000	2506783020	5,725,032	164	85,568	*Hutchinsia alpina*	France	3
*Pseudomonas syringae* CC1524	AVEK00000000	2506783021	5,828,366	264	44,224	Stream water	France	4
*Pseudomonas syringae* CC1629	AVEE00000000	2524614586	5,932,928	261	75,208	Oats	USA	4
*Pseudomonas syringae* CC1630	AVED00000000	2524614587	6,056,064	283	52,218	Sainfoin	USA	4
*Pseudomonas syringae* USA011	AVDX00000000	2509276053	6,429,288	198	91,862	Stream water	USA	4

a Projects have been deposited at GenBank under these accession numbers, but the version described in this paper is version XXXX02000000.
